# The effect of a mindfulness-based stress intervention on neurobiological and symptom measures in adolescents with early life stress: a randomized feasibility study

**DOI:** 10.1186/s12906-021-03295-1

**Published:** 2021-04-15

**Authors:** Zsofia P. Cohen, Kelly T. Cosgrove, Elisabeth Akeman, Sara Coffey, Kent Teague, Jennifer Hays-Grudo, Martin P. Paulus, Robin L. Aupperle, Namik Kirlic

**Affiliations:** 1 Laureate Institute for Brain Research, 6655 S Yale Avenue, Tulsa, OK 74136 USA; 2 Department of Psychology, University of Tulsa, 800 S Tucker Drive, Tulsa, OK 74104 USA; 3 Center for Health Sciences, Oklahoma State University – Tulsa, 1111 W 17th St, Tulsa, OK 74107 USA; 4 Departments of Surgery and Psychiatry, University of Oklahoma School of Community Medicine, 4502 A 41st St, Tulsa, OK 74135 USA; 5 Oxley College of Health Sciences, School of Community Medicine, University of Tulsa, 1215 S Boulder Ave W, Tulsa, OK 74119 USA

**Keywords:** Adolescence, Early life stress, Inflammation, Cortisol, Epigenetic, Mindfulness, Prevention, Resilience

## Abstract

**Background:**

Early life stress (ELS) has been linked to poor mental and physical health outcomes in adolescence and adulthood. Mindfulness reduces symptoms of depression and anxiety and improves cognitive and social outcomes in both youth and adults. However, little is known whether mindfulness can mitigate against the adverse neurobiological and psychological effects of ELS. This study aimed to examine the feasibility of conducting a group mindfulness intervention in adolescents with ELS and provide preliminary indication of potential effects on stress-related biomarkers and mental health symptoms.

**Methods:**

Forty adolescents were randomized to receive either eight sessions of Mindfulness-Based Stress Reduction for Teens in group format (MBSR-T; *n* = 21) or Treatment as Usual Control group (CTRL; *n* = 17). Outcomes were assessed at baseline and follow-up and included measures associated with neurobiological functioning (immune and endocrine biomarkers) and self-reported mental health (depressive) symptoms. Linear mixed effects models were used to assess the effects of group and time on these outcome measures.

**Results:**

Sixteen of the 21 adolescents completed the intervention, attending an average of 6.5 sessions. The model examining cortisol responses to stress induction revealed medium effects trending toward significance (Cohen’s *d* = .56) for anticipatory cortisol levels in the MBSR-T relative to CTRL groups. No significant effects were found in models examining C-reactive protein or interleukin 6 inflammatory markers. The model examining depressive symptoms revealed a medium effect for symptom reduction (Cohen’s *d* = .69) in the MBSR-T relative to CTRL groups.

**Conclusions:**

This study demonstrated feasibility of conducting a group-based MBSR-T intervention for adolescents with ELS. There was some evidence for efficacy on a symptom level with potential subtle changes on a biological level. Future larger studies are needed to determine the efficacy of group-based mindfulness interventions in this population.

**Trial registration:**

Identifier #NCT03633903, registered 16/08/2018.

**Supplementary Information:**

The online version contains supplementary material available at 10.1186/s12906-021-03295-1.

## Introduction

Early life stress (ELS) is characterized by chronic emotional and physical abuse and neglect, sexual abuse, parental psychopathology and substance abuse, and household dysfunction (e.g., parental incarceration, interpersonal violence). An estimated 678,000 American youth (birth through age 18) are victims of abuse and neglect annually in the United States, with Child Protective Services referrals involving 7.8 million children in 2018 [1]. Over the last half-decade, the estimated lifetime direct and indirect costs (e.g., medical, productivity loss, criminal justice) of child abuse and neglect per non-fatal victim has increased from $210,000 to $831,000 [2, 3], further highlighting ELS as a public health crisis.

ELS has been linked to poorer mental and physical health outcomes in adolescence and adulthood. It accounts for nearly half of all childhood-onset mental health disorders and one third of adult-onset disorders [4, 5]. ELS significantly elevates the risk for mood and anxiety disorders [6–9], externalizing and substance use disorders [10–12], personality disorders [13–15], as well as suicidal ideation and attempts [16, 17]. ELS-exposed individuals evidence poorer psychotherapy and pharmacotherapy outcomes relative to their non-exposed treatment-receiving counterparts [18]. As a large portion of extant literature focuses on adults, indicating the long-term effects of ELS, there is a need to develop interventions optimized for youth with ELS that can help mitigate their potential for negative mental and physical health outcomes.

The consequences of ELS are complex and are believed to stem from alterations in a number of neurobiological processes involved in generation and regulation of emotional responses, including endocrine, immune, epigenetic, and brain circuits [19–23]. ELS has been shown to disrupt the function of the hypothalamic-pituitary-adrenal (HPA) axis, which plays a prominent role in stress response and regulation [24–30], as well as downstream inflammatory [31–37] and epigenetic (e.g., FK506 binding protein 5 [FKBP5]) changes [38–42]. While childhood and adolescence are stages of particular vulnerability to psychopathology [43], proximity to ELS exposure, increased plasticity, and ongoing development provide an opportunity for normalization in systems subservient to stress responses and emotion regulation as a result of intervention, as well as increased resilience [44, 45]. A number of psychological interventions (e.g., Cognitive-Behavioral Therapy [CBT], Trauma-Focused CBT, Prolonged Exposure Therapy, Cognitive Processing Therapy, Parent Child Interaction Therapy, Child-Parent Psychotherapy, and Emotion Regulation approaches) have been efficacious in reducing symptoms of depression, anxiety, or posttraumatic stress disorder (PTSD) in ELS-exposed youth [46]. However, it remains unclear whether, and to what extent, interventions exert influence on the disrupted underlying neurobiological mechanisms involved in the response to and regulation of stress.

Psychological techniques that promote emotional awareness and regulation are well-suited to target mechanisms involved in responses to psychological stressors and thus may be particularly beneficial for ELS-exposed youth in reducing current symptoms and increasing resilience [47–49]. Mindfulness practice offers one such approach, such that it encourages the development of awareness of one’s thoughts, emotions, and behaviors by increasing the ability to observe and direct internal experiences [50]. Research involving both adults and youth shows that mindfulness reduces symptoms of depression and anxiety and improves cognitive and social outcomes [47–51]. Moreover, recent data indicate that mindfulness practice may positively influence processes involved in regulation of stress responses in adults, including changes in expression of pro-inflammatory genes [52] and methylation of FKBP5 in adults with PTSD [53], immune and endocrine system markers (e.g., reduction in C-reactive protein and increased cortisol reactivity, respectively [54, 55]), and activation in a distributed network of brain regions involved in interoception, self-referential processing, threat detection, and emotion regulation [46, 56–58]. Therefore, mindfulness represents a potential regulatory intervention that may inhibit or reverse some of the deleterious long-term effects of ELS exposure. However, the efficacy of a mindfulness-based intervention for youth exposed to ELS has not been investigated.

The present study aimed to address this gap in the literature by determining the feasibility of a group mindfulness intervention, Mindfulness-Based Stress Reduction for Teens (MBSR-T), for adolescents exposed to ELS. We examined treatment completion rates and average number of sessions attended to assess feasibility. To provide preliminary data concerning the impact of MBSR-T on biological and clinical outcomes, we assessed changes from pre to post-treatment in cortisol reactivity in response to a psychosocial stressor and immune system function (primary outcomes), symptoms of depression (secondary outcome), and expression of HPA axis regulatory gene FKBP5, incidence of substance use, and self-reported mindfulness and resilience traits (exploratory outcomes).

## Methods

### Participants

**Fig. 1:**
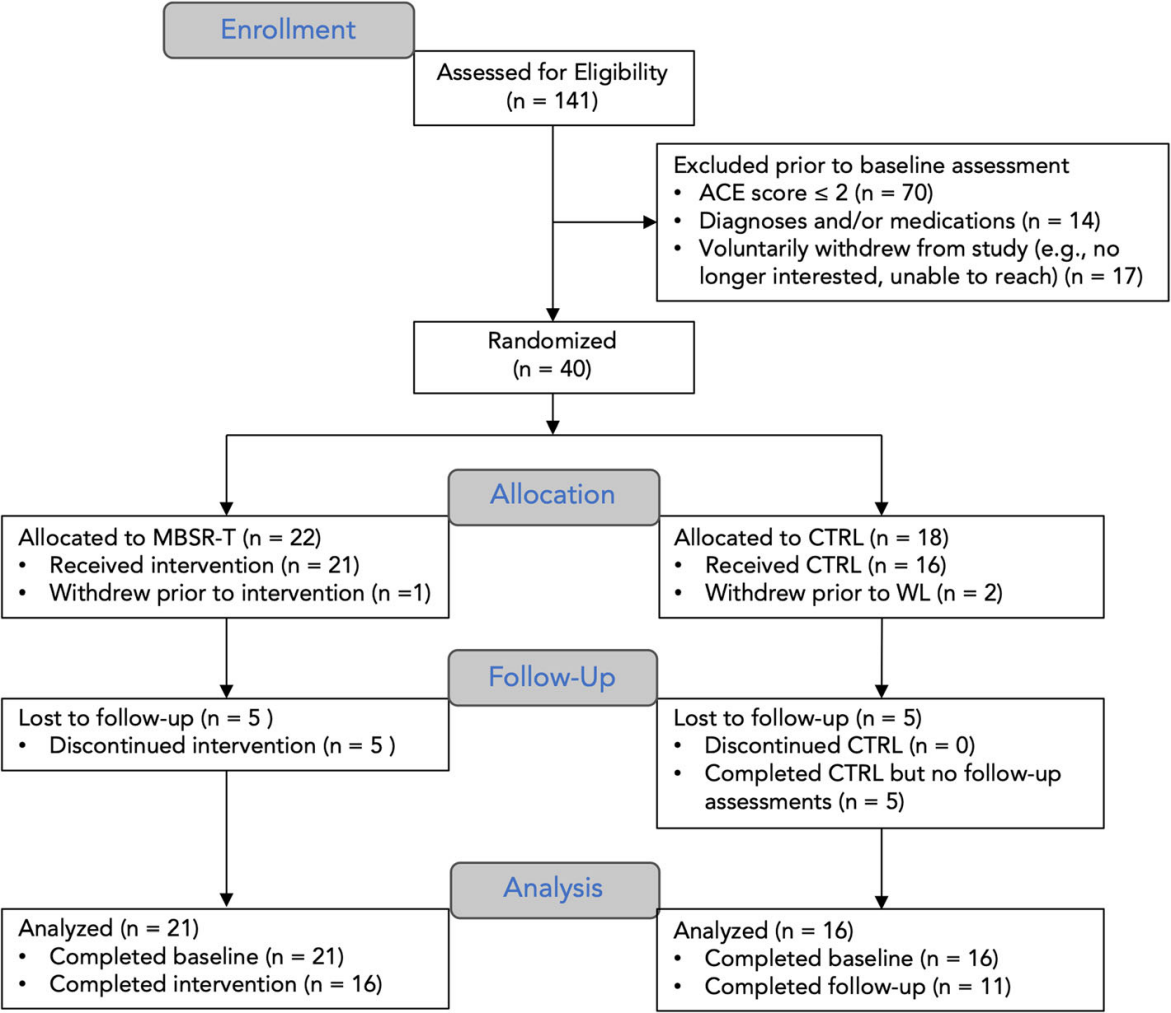
CONSORT diagram. Figure shows the flow of adolescents through the phases of the study and the number that withdrew at each time point. Abbreviations: ACE, Adverse Childhood Experiences; CTRL, Control; MBSR-T, Mindfulness Based-Stress Reduction for Teens

The present feasibility study used a parallel randomized controlled trial design. Adolescents and their families were recruited from the community using flyers, radio advertisements, billboards, and a school-based messaging platform (e.g., PeachJar). All data collection took place at a midwestern private research institute. A total of 141 adolescents were assessed for eligibility via a phone screening in which caregivers reported the number of Adverse Childhood Experiences (ACEs [59]) the adolescent had experienced. Those with three or more parent-reported ACEs were included in the study (≥3 ACEs has been associated with greater impairments [60]). Exclusion criteria were kept to a minimum in order to increase generalizability; however, neurological and psychotic disorders and active suicidal ideation were deemed exclusionary. Psychotropic medications were permitted so long as participants had been on a stable (i.e., unchanged) dose for six weeks or longer. A total of 40 adolescents (age: 14.3 [.76]; 59% male) were randomized (allocation ratio 1:1) to either Mindfulness-Based Stress Reduction for Teens (MBSR-T) or Control (CTRL). The Consolidated Standards of Reporting Trials (CONSORT) diagram is provided in Fig. 1. Parents and adolescents provided written informed consent and assent, respectively. Adolescents were compensated for baseline and follow-up assessment visits, as well as for completing surveys at each time point, but were not compensated for completing the mindfulness intervention. Research was approved by the Western Institutional Review Board and conducted in accordance with the Declaration of Helsinki. The study was registered at the US National Institutes of Health (ClinicalTrials.gov identifier #NCT03633903, registered 16/08/2018). No changes were made to methods or trial outcomes after enrollment commenced.

### Procedures

Baseline and follow-up assessments included collection of biological samples, a stress-induction task, and a range of self-report measures. At baseline, adolescents completed measures assessing mental health symptoms and traits, as well as a stress-induction task. Adolescents also provided blood samples on a separate day. In the weeks preceding allocation, selected symptom measures were completed electronically to better capture baseline mental health symptoms and traits. Adolescents were then randomly assigned to one of the two conditions, Mindfulness-Based Stress Reduction for Teens (MBSR-T) intervention or no-treatment control (CTRL). Block randomization was conducted by the principal investigator (NK) via random number generator and was not blinded to either participants or investigators at the time of enrollment. Enrollment, assignment, and data collection were undertaken by the study coordinator (EA). MBSR-T commenced within two weeks of baseline assessment and consisted of eight sessions over four weeks. Prior to each session, MBSR-T participants completed brief symptom and treatment compliance assessments. The CTRL group completed the symptom assessments online. Following the conclusion of four weeks, follow-up assessments (i.e., repeated baseline measures) were completed by both groups. Primary outcomes included changes in cortisol and inflammatory markers, while secondary outcomes focused on symptoms depression. Exploratory outcomes assessed changes in HPA axis gene expression, mindfulness, resilience, and substance abuse. All outcome measures were collected at baseline and follow-up, with symptoms of depression (i.e., Mood and Feelings Questionnaire-Short Form [MFQ-SF [61]]) and suicidality (Suicide Behavior Questionnaire-Revised [SBQ-R [62]]) collected at each session timepoint for all subjects. The Columbia Suicide Severity Rating Scale (C-SSRS [63];) was administered to subjects who endorsed any suicidal ideation at any timepoint. Symptoms of depression and suicidal thoughts and behaviors were used to assess participant safety, while the Working Alliance Inventory (WAI [64];) assessed the therapeutic bond between participants and therapists. A contingency safety plan was put in place to address any emergent safety concerns. Blood samples were taken by a trained phlebotomist and participants were monitored for pain, dizziness, as well as bruising and infection at the puncture site.

### Intervention

#### Mindfulness-based stress reduction for teens

The active intervention, MBSR-T, was provided in a group format [51, 65]. Relative to MBSR [66], MBSR-T has been slightly modified for use with adolescents ages 13–18 and takes into account the attentional capabilities of youth and the impact of technology on interpersonal interactions. Adaptations include shortened formal mindfulness practice (10–20 min, rather than 40 for adults), On-Your-Own-Practices (rather than homework), and no day-long retreat [51, 65]. Sessions were conducted twice a week for four weeks to further examine feasibility and reduce participant burden. Other brief mindfulness interventions (e.g., 2–5-week programs) have been successfully implemented [67–69] with similar gains in buffering stress reactivity [70].

Topics of focus included intention (direction of effort toward mindfulness practice), attention (experiencing what is taking place in the present moment), and attitude (nonjudgmental attributions of cognitive, emotional, and somatic experiences), with each session having specific foci. Session 1 centered on examining and defining the foundations of stress and providing an introduction to mindfulness (mindfulness practice: mindful eating; dropping-in mindfulness). Session 2 explored the effect of stress on the mind and body, as well as beginning a personal mindfulness practice (mindfulness practice: body scan mindfulness). Session 3 focused on developing and strengthening mindfulness practice, including learning how to increase present-moment awareness (mindfulness practice: mindful breathing; sitting mindfulness). Session 4 centered on cultivating self-care and facilitating awareness of positive experiences and pleasant moments (mindfulness practice: mindful walking and movement; heartfulness mindfulness). In Session 5, mindfulness exercises were used to notice, be, and work with thoughts, as well as to facilitate awareness of negative experiences and unpleasant moments (mindfulness practice: yoga and mindful movement; mindful stopping). Session 6 further focused on improving awareness through mindfulness and use of positive coping strategies and behaviors to manage life’s events (mindfulness practice: sitting mindfulness; mindful homework and test taking). Session 7 cultivated mindfulness resilience and building mindful relationships (mindfulness practice: mindful gratitude taking; body scan mindfulness). Finally, Session 8 focused on reviewing the MBSR-T program and making mindfulness a continuing part of daily life (mindfulness practice: dropping in mindfulness; gratitude practice).

Adolescents assigned to MBSR-T were given a workbook to use for On-Your-Own-Practice assignments. The study investigator and a doctoral student in clinical psychology supervised by the study investigator delivered the intervention. The study investigator, a licensed health service psychologist, completed a 12-session trainers’ training on MBSR-T prior to study commencement. Sessions were video recorded. A board-certified child and adolescent psychiatrist trained in mindfulness (SC) listened to the tapes and provided weekly supervision to promote treatment fidelity and a developmentally appropriate intervention, while also assessing for potential bias introduced by intervention facilitators.

#### Control condition

Adolescents in the CTRL group were followed for the duration of the study with self-report measures administered at the same time intervals as the MBSR-T groups. Participants were not asked to stop any treatments or activities they were already undergoing, including psychotherapy, pharmacotherapy, or other services during the duration of the study. All participants were provided with a list of community-based referrals upon randomization.

### Measures

#### Self-report measures

For an administration schedule of measures, please see Table S1. All of the self-report measures have been found to be psychometrically reliable and valid. All measures, with the exception of the caregiver reports of ACEs, were reported by adolescents. The ACEs scale [59] assessed instances of physical, sexual, and emotional abuse; physical and emotional neglect; and household dysfunction (divorce, parental mental illness, domestic violence, parent incarceration). The scale contains 10 questions rated on a yes-no basis. A composite ACEs score was calculated by totaling all instances of an affirmative answer across caretaker (screening) and adolescent reports (baseline and follow-up). Additionally, the Childhood Trauma Questionnaire (CTQ [71]) provided a measure of severity of exposure to childhood trauma. The CTQ comprises of five subscales, including physical abuse, sexual abuse, emotional abuse, physical neglect, and emotional neglect. The CTQ contains 25 questions (5 each of the aforementioned subscales) as well as an additional 3 questions to assess denial. Items are rated on a 5-point Likert scale (0 = never true, 5 = very often true) with scores ranging between 25 to 125.

Adolescent depressive symptoms were measured using the Mood and Feelings Questionnaire-Short Form (MFQ-SF [61]). This 13-item questionnaire is derived from the DSM-III-R criteria for depression and assessed phrases regarding how the subject has been feeling or acting. Items are rated on a 3-point Likert scale (0 = not true, 1 = sometimes, 2 = true), with scores ranging from zero to 26. The Adolescent Alcohol and Drug Involvement Scale (AADIS [72]) measured adolescents’ history of and/or current substance use, potential interference with life, and stigma. First, 13 substances are rated on an 8-point scale for frequency of use (0 = never used, 7 = several times a day), with scores ranging from zero to 84 for frequency of total use. Further questions about most recent use and usage effects are then asked. Examples of these items include ‘How do you get your alcohol or drugs?’ and ‘Why did you take your first drink or first use drugs?’ The Connor-Davidson Resilience Scale, 10 item (CD-RISC 10 [73]) assessed stress coping abilities. Scores range from zero to 40, with lower indicating lower levels of resilience. The Mindful Attention Awareness Scale – Adolescent (MAAS-A [74]) measured intrapersonal awareness across 14 items (1 = almost always, 6 = almost never), with scores ranging from 14 to 84. Notably, the MAAS-A has been found to be sensitive to change in mindfulness as a result of topics covered in mindfulness interventions [75].

Finally, the MBSR-T group alone completed the Homework Rating Scale (HRS [76]) to assess between-session compliance with intervention materials. Twelve items were rated (0 = none, 4 = all) for score ranges of zero to 48. MBSR-T participants also completed the Working Alliance Inventory for Children and Adolescents (WAI-CA [64]) to assess therapeutic agreement on goals and tasks of therapy, as well as the development of affective bond. A total score is also derived. Scores range from four to 20 for each subscale and 12–60 for the total scale score, with higher scores indicating greater alliance.

#### Stress induction task

We used the Trier Social Stress Test for Children (TSST-C [77, 78]) to assess biological responses to stressful situations. The TSST-C consisted of public speaking and mental arithmetic serial subtraction components, with different prompts for the two timepoints. Research confederates not otherwise involved with the study and data collection delivered the TSST-C. Baseline and follow-up TSST-C administration involved a different pair of associates. Prior to beginning and upon completion of the TSST-C, participants rated how they currently felt on a five-point Likert scale (very calm to very anxious). For the public speaking component, adolescents were given the stem of a story and asked to complete the story in an interesting and exciting way. Further, they were told that the story ending should be better than those provided by other participants. The participant then had a five-minute preparation period, followed by a five-minute presentation to two associates. If finished prior to the five-minute story period, adolescents were prompted to continue until the time elapsed. Directly following this, a serial subtraction task (e.g., subtracting by 13 from 1023 [baseline]; subtracting by 17 from 1027 [follow-up]) was completed. This task was also completed over a five-minute period in front of the associates. Adolescents were encouraged to work as quickly and accurately as possible and were asked to restart if errors were made. Salivary cortisol was collected prior to TSST-C and 10 min following the completion of the TSST-C (i.e., 20 min from the TSST-C stress induction).

#### Biological samples

Saliva was collected using passive drool SalivaBio collection tubes (Salimetrics) and stored at -30 °C. Saliva was then centrifuged, aliquoted, and stored at -80 °C. Cortisol concentrations in saliva samples was determined using Salimetrics High Sensitivity Salivary Cortisol enzyme-linked immunosorbent assay (ELISA) kits. Blood was collected using standard venipuncture procedures with BD vacutainer tubes, cell preparation tubes with sodium citrate for Peripheral Blood Mononuclear Cell (PBMC) isolation, and serum vacutainer tubes with clot activator. A total of 25 mL of blood was drawn during the entire study by a trained phlebotomist. PBMCs were aliquoted and stored in liquid nitrogen and serum was isolated and stored at -80 °C. Interleukin 6 (IL-6) and C-reactive protein (CRP) levels in the serum samples were assayed using MesoScale Discovery V-PLEX assay kits using the MESO QuickPlex SQ 120. Ribonucleic acid (RNA) was isolated from PBMCs using Qiagen RNeasy Micro kits and a complimentary deoxyribonucleic acid (cDNA) bank was created using Omniscript kits. Gene expression analyses were completed by quantitative real-time polymerase chain reaction (qRT-PCR) using the QuantStudio 12 K Flex Real-Time PCR System for a specific gene of interest (i.e., FKBP5) and one control gene (i.e., GAPDH). Saliva samples were collected between 9:30–18:00, whereas blood samples were collected prior to 15:00.

### Data analysis

The primary analysis approach was between-group differences from baseline to follow-up. The report of effects in this study focuses on effect sizes (Cohen’s d) due to the feasibility nature of the study. Several measures were normalized to account for outliers (i.e., all biological variables, AADIS, Body Mass Index, and CTQ). All statistical analyses were performed using R programming environment [79]. Descriptive statistics were obtained using the R package ‘psych’ [80] and independent samples t-tests examined group differences on demographic variables.

Linear regressions were used to evaluate relationships between baseline biological variables and ELS severity. Gender was used as a covariate in all regression analyses. For analyses involving biomarkers and genes, body mass index (BMI) was added as a covariate. Finally, for cortisol regression analyses, wake-up time and time of cortisol collection were included as covariates. To examine changes in biological variables and self-reported mental health symptoms as a function of treatment, linear mixed effects models (LMEs) were conducted using the ‘lmer’ function in R package ‘lme4’ [81] and plots were generated with ‘emmeans’ [82]. Fixed effects included group and time. Random effects included subject. Follow-up pairwise comparisons were conducted using estimated marginal means to further describe the effects of group and time on the outcome variables. Baseline MFQ scores for analysis were calculated by averaging responses across three timepoints (e.g., baseline, online assessments 1 and 2).

### Sample size justification

According to Julious [83], a sample size of 12 per group is recommended for feasibility randomized clinical trials. A sample size of 20 per group, allowing for a conservative 20% dropout, would give us *N* = 16 total per group, which exceeded the threshold for a sufficiently precise estimate of variance in continuous variables to use in future studies. Furthermore, with *N* = 20 per group and 20% attrition, we were 80% powered to detect medium size effects (*f* = .25) between groups from baseline to follow-up on continuous variables of interest.

## Results

### Descriptive analyses

A baseline sample of 40 adolescents was enrolled and randomized to MBSR-T or CTRL conditions. Recruitment continued throughout the enrollment of the target sample size of 40 adolescents. Follow-up data was collected within two weeks of the completion of MBSR-T or CTRL allocation. Participants ranged from ages 13–15, with a mean age of 14.3 and a standard deviation of 0.9. The sample was 59% male. The MBSR-T and CTRL groups did not differ in terms of age, sex, race, ethnicity, ELS-exposure histories, medication usage, connection to psychotherapy resources, or parental psychopathology (*p*s > .05). Demographic and clinical characteristics across groups are reported in Table 1. The regression model including ACEs, gender, and BMI explained 34% of the variance in CRP levels [*F*_(3, 27)_ = 6.97, *p* < .005, *f*^2^ = .46], with BMI (*β* = .64) as the only significant predictor. Similarly, the regression model including ACEs, gender, and BMI explained 36% of the variance in IL-6 levels [F _(3, 27)_ = 6.49, *p* < .005, *f*^2^ = .55], with BMI (*β* = .65) as the only significant predictor. Full results for regression models are available in Table S2.

**Table 1:** Baseline demographics and clinical characteristics of the mindfulness-based stress reduction for teens and control groups

Characteristics	MBSR-T (*n* = 21)		CTRL (*n* = 17)		Group Differences	
	Mean	SD	Mean	SD	t	p
Age	14.33	0.73	14.29	0.77	0.16	0.87
ACEs	5.90	1.70	6.11	2.03	−0.35	0.73
CTQ	46.24	12.45	49.41	11.00	−0.82	0.42
Emotional Abuse	9.81	4.49	11.06	4.88	−0.82	0.42
Emotional Neglect	10.24	4.76	10.53	4.21	−0.20	0.84
Physical Abuse	7.71	3.07	7.18	2.19	0.61	0.55
Physical Neglect	8.24	3.10	8.59	3.71	−0.32	0.75
Sexual Abuse	5.33	0.86	6.94	4.48	−1.61	0.12
	**N**	**%**	**N**	**%**	**χ2**	**p**
Sex					0.79	0.38
Male	14	67	8	47		
Female	7	33	9	53		
Ethnicity					1.26E-30	1.0
Hispanic	2	10	1	6		
Non-Hispanic	19	90	16	94		
Race					6.99	0.22
White	10	48	9	53		
American Indian or Alaska native	2	10	2	12		
Black or African American	4	19	5	29		
Asian or Pacific Islander	0	0	0	0		
More than one race	5	24	0	0		
"Other,” unspecified	0	0	1	6		
Mental health diagnoses	7	33	4	24	0.90	0.76
Psychotropic medication use	6	29	6	35	0.01	0.93
Psychotherapy	7	33	4	24	0.05	0.94
Parental mental health diagnoses	9	43	9	53	0.09	0.77

Note. Percentages are rounded to the nearest whole percent

*Abbreviations*: *ACEs* Adverse Childhood Experiences, *CTQ* Childhood Trauma Questionnaire, *CTRL* Control, *MBSR-T* Mindfulness Based-Stress Reduction for Teens

### MBSR-T feasibility

A total of three groups completed the MBSR-T program (July–August 2018; October–November 2018; January–February 2019), ranging between five and nine adolescents per group. Twenty-two participants were randomized to MBSR-T. One subject withdrew prior to completing baseline procedures. Thus, 21 participants allocated to MBSR-T began the intervention. Sixteen of the 21 participants allocated to MBSR-T completed the intervention, with an average attendance of 6.5 sessions. One subject never started the intervention, two dropped out after attending one session, and two dropped out after attending two sessions. Reasons cited for participant withdrawal included a loss of interest (*N* = 3), three consecutive missed sessions during the intervention (*N* = 1), or scheduling conflicts (*N* = 1). On average, MBSR-T participants (*N* = 16) were moderately compliant with On-Your-Own-Practices (i.e., homework assignments; Table S3). With respect to working alliance, MBSR-T participants reported that they very often agreed with goals and tasks of therapy, as well as developed an affective bond with therapists (Table S4). Working Alliance saw minimal increases across each subscale over time, including a small, albeit not statistically significant, effect increase across training for total scores (*F*_(6, 84)_ = 1.20, *p* = .31, *f* = .12; Fig. S1). No adverse events were observed or reported during the intervention.

### Outcome analyses

#### Biological variables

**Fig. 2:**
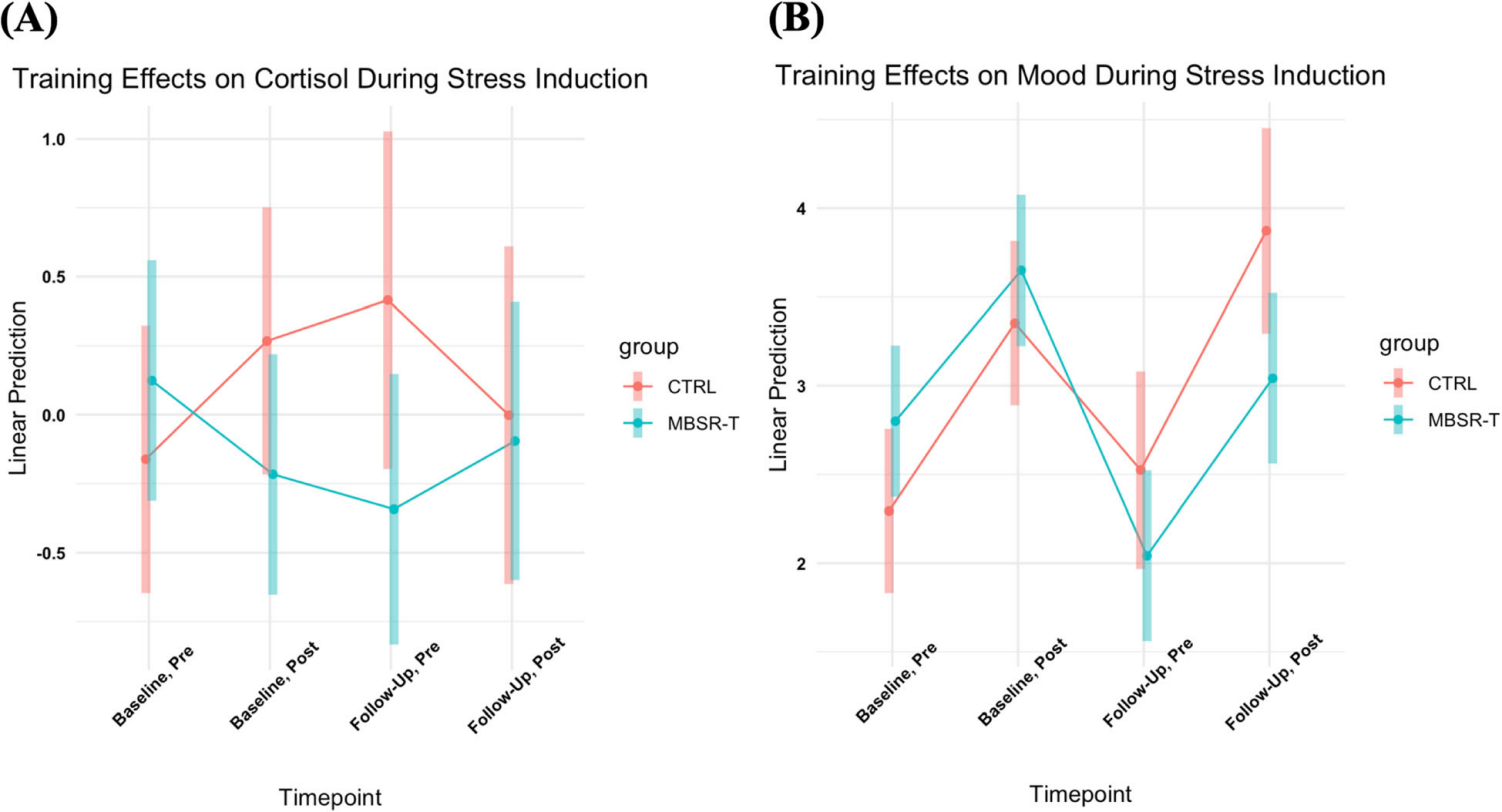
**a** The trajectory of the self-reported mood scores before and after the Trier Social Stress Task for Children (TSST-C). Participants were asked to rate their mood on a 5-point Likert scale (1 = very calm, 5 = very anxious). Participants completing the mindfulness intervention evidenced reduced anxious arousal before and after the TSST-C when compared with participants assigned to treatment as usual. **b** The trajectory of cortisol response before and after the Trier Social Stress Test  for Children (TSST-C). Participants completing the mindfulness intervention evidenced reduced cortisol levels prior to the TSST-C commencing when compared with participants assigned to control group. Abbreviations: CTRL, Control; MBSR-T, Mindfulness Based-Stress Reduction for Teens

Full statistical results for LMEs are provided in Table 2. Our primary interest was in the Group by Time interaction analyses. LMEs revealed a small non-significant effect for Group by Time interaction for CRP (*F*_(1, 26)_ = .77, *p* = .39, *d* = −.34), while no effect was observed for IL-6 (*F*_(1, 22)_ = .004, *p* = .98, *d* = −.03). Conversely, LME analysis examining cortisol response to stress induction evidenced a medium effect trending toward significance for the Group by Time interaction (*F*_(3, 86)_ = 2.36, *p* = .077, *d* = .60; Fig. 2a). Pairwise comparisons revealed a medium effect size trending toward significance in anticipatory cortisol levels preceding the social stress task at follow-up for MBSR-T relative to CTRL participants [*t*_(111)_ = 1.92, *p* = .058, *d* = − .56]. Similarly, LME analysis examining self-reported anxious arousal to stress induction revealed a large significant effect for the Group by Time Interaction (*F*_(3, 86)_ = 4.49. *p* = .006, *d* = .83; Fig. 2b). Follow-up pairwise comparisons revealed a medium significant effect size reduction in self-reported anxiety following the TSST-C at follow-up for MBSR-T relative to CTRL participants [*t*_(108)_ = 2.19, *p* = .031, *d* = −.74].

**Table 2:** Unadjusted means, standard deviations, effect sizes, and main analyses of change from baseline to follow-up in mindfulness-based stress reduction for teens and control groups

**Primary Outcomes**	**Mean and Standard Deviation**				**Statistic**					
	**MBSR-T (*n* = 21)**		**CTRL (*n* = 17)**		**Effect**	**Cohen’s d**	**Estimate**	**SE**	**t**	**p**
CRP					Interaction	−0.34	−0.35	0.4	− 0.88	.39
Baseline	1.16	3.82	2.39	5.75						
Follow-up	0.83	1.45	0.99	1.32						
IL-6					Interaction	−0.03	−0.02	0.38	−0.07	.95
Baseline	0.47	0.48	0.83	1.19						
Follow-up	0.46	0.32	0.66	0.65						
Cortisol										
Baseline, Pre	0.12	0.89	−0.16	1.15						
Baseline, Post	−0.22	0.88	0.27	1.10	Interaction	0.45	0.77	0.38	2.02	<.05
Follow-up, Pre	−0.21	1.054	0.34	0.85	Interaction	0.50	1.04	0.44	2.37	<.05
Follow-up, Post	0.05	1.16	−0.08	0.76	Interaction	0.18	0.38	0.44	0.86	.40
Stress										
Baseline, Pre	2.80	1.11	2.29	0.69						
Baseline, Post	3.65	0.99	3.35	0.70	Interaction	0.13	0.21	0.36	0.58	.56
Follow-up, Pre	2.13	1.19	2.55	0.82	Interaction	0.52	0.99	0.41	2.42	<.05
Follow-up, Post	3.13	1.13	3.90	0.88	Interaction	0.68	1.34	0.42	3.22	<.01
**Secondary Outcomes**	**Mean and Standard Deviation**				**Statistic**					
	**MBSR-T (*n* = 21)**		**CTRL (*n* = 17)**		**Effect**	**Cohen’s d**	**Estimate**	**SE**	**t**	**p**
Depression					Interaction	0.69	2.71	1.49	1.82	.08
Baseline	6.81	4.53	9.39	6.54						
Follow-up	3.82	4.61	8.17	8.07						
**Exploratory Outcomes**	**Mean and Standard Deviation**				**Statistic**					
	**MBSR-T (*n* = 21)**		**CTRL (*n* = 17)**		**Effect**	**Cohen’s d**	**Estimate**	**SE**	**t**	**p**
Substance Use										
All					Interaction	0.17	0.13	0.25	0.51	.61
Baseline	1.48	1.91	3.12	5.17						
Follow-up	1.52	2.69	3.59	7.62						
Alcohol					Interaction	0.51	0.4	0.28	1.41	.17
Baseline	0.71	0.85	0.59	1.06						
Follow-up	0.78	1.00	1.00	1.28						
Marijuana					Interaction	0.86	0.53	0.23	2.34	<.05
Baseline	0.57	1.29	0.59	1.06						
Follow-up	0.50	1.34	1.08	1.68						
Mindfulness					Interaction	0.21	2.41	3.95	0.61	.55
Baseline	53.83	9.92	49.47	7.25						
Follow-up	58.12	12.39	55.75	15.73						
Resilience					Interaction	0.04	0.19	1.94	0.1	.92
Baseline	25.1	6.67	25.18	6.87						
Follow-up	25.82	7.58	27.75	8.30						
FKBP5					Interaction	0.26	0.29	0.44	0.65	.52
Baseline	5.26	0.85	5.35	0.78						
Follow-up	5.25	1.22	5.58	0.52						

Note. Stress is the self-reported anxious arousal prior to and after the stress induction task. Cortisol is measured in μg/dL. IL-6 is measured in pg/mL. CRP is measured in mg/L.

*Abbreviations*: *CRP* C-reactive protein, *CTRL* Control, *IL-6* interleukin-6, *MBSR-T* Mindfulness Based-Stress Reduction for Teens

#### Depression

**Fig. 3:**
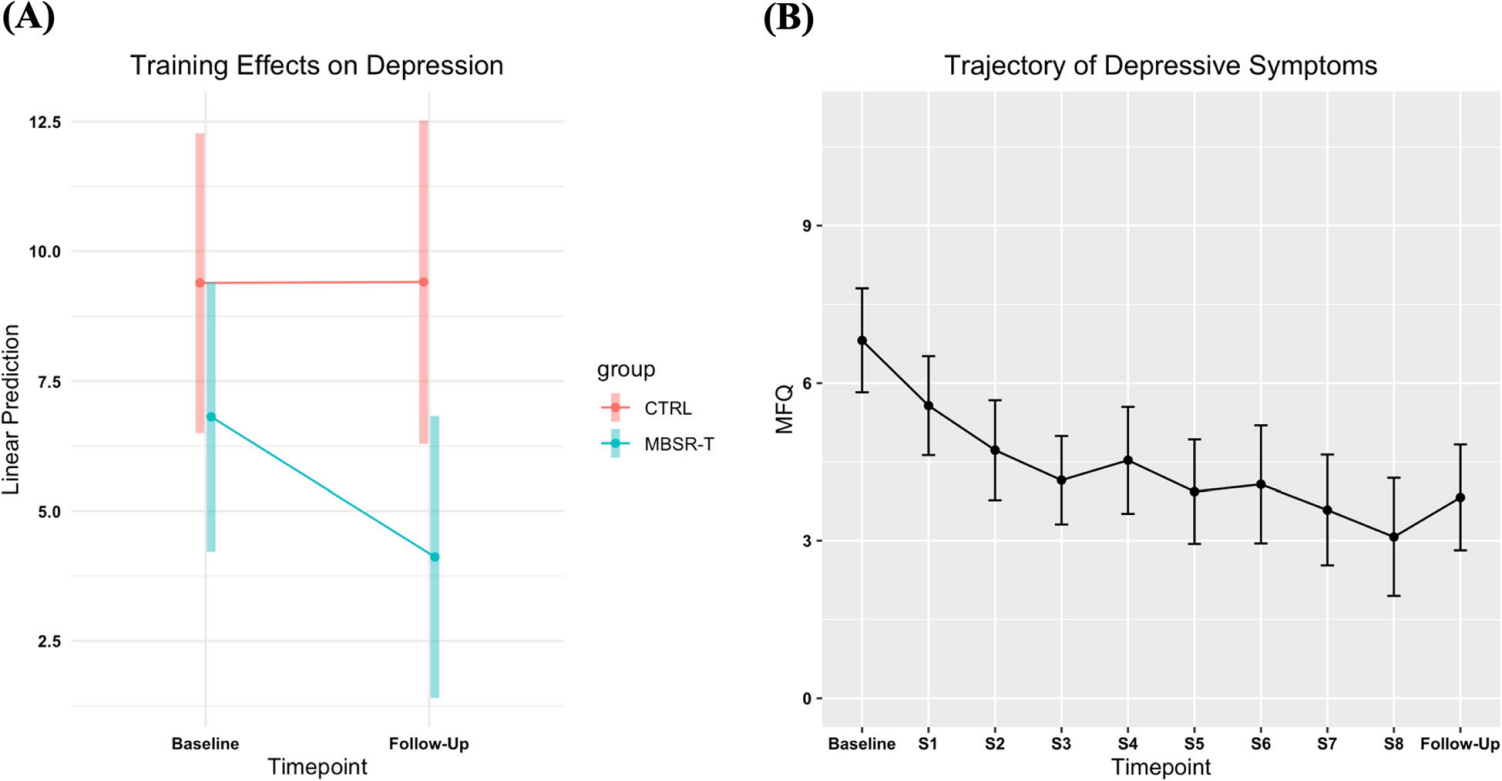
Change in depression by group over time. Shown here are interaction plots of (**a**) estimated marginal means based on the fitted linear mixed-effects model for depression from baseline to follow-up between groups and (**b**) means and standard deviations based on the fitted linear mixed-effects model for depression across treatment within the MBSR-T group. The MBSR-T group showed a significant decrease in depressive symptoms from baseline to follow-up, whereas the CTRL group did not. Further, a decreasing trend was observed over the course of treatment. Abbreviations: CTRL, Control group; MBSR-T, Mindfulness Based-Stress Reduction for Teens; MFQ, Mood and Feelings Questionnaire; S, Session

LMEs for depression symptoms revealed a medium to large effect trending toward significance for the Group by Time interaction [*F*_(1, 28)_ = 3.31, *p* = .079, *d* = .69; Fig. 3a). Follow-up pairwise comparisons revealed a large significant effect size reduction in symptoms of depression from baseline to follow-up for MBSR-T [*t*_(28.2)_ = − 2.972, *p* < .01, *d* = 1.40], while no effect was observed in the CTRL group [*t*_(29)_ = − .016, *p* = .988, *d* = .01]. For the MBSR-T group, a medium significant effect size was evidence for continuous decrease in symptoms of depression over the course of treatment (*F*_(9,139)_ = 5.27, *p* < .001, *d* = .51; Fig. 3b).

#### Exploratory analyses

A large significant effect was identified for the Group by Time interaction for marijuana use [*F*_(1, 29)_ = 5.47, *p* = .03, *d* = .86]. Follow-up pairwise comparisons revealed a large significant effect size increase in marijuana use in the CTRL group from baseline to follow-up [*t*_(29)_ = 2.414 *p* = .02, *d* = 1.40], whereas a small non-significant effect size decrease in marijuana use was observed within the MBSR-T group [*t*_(29)_ = − .74, *p* = .47, *d* = .24]. Small and medium non-significant Time by Group effects were found for the AADIS total score [*F*_(1, 36)_ = 0.26, *p* = .61, *d* = .17] and the alcohol subscale [*F*_(1, 31)_ = 1.98, *p* = .17, *d* = .51], respectively. Small non-significant Group by Time interaction effects were found for mindfulness [*F*_(1, 32)_ = 0.37, *p* = .55, *d* = .21] and HPA axis regulating gene FKBP5 [*F*_(1, 25)_ = 0.42, *p* = .52, *d* = .26]. Group by Time interaction effect sizes were not found for the measure of resilience [*F*_(1, 32)_ = 0.01, *p* = .55, *d* = .04].

## Discussion

This study aimed to examine the feasibility of a group mindfulness intervention for adolescents with ELS, and to provide a preliminary indication of effects on stress-related biomarkers and mental health symptoms. Results yielded two main results. First, the mindfulness training appeared to be an acceptable, safe, and feasible intervention for the population of interest. Second, MBSR-T showed promise in reducing symptoms of depression and marijuana use, as well as self-reported anxiety and cortisol response during stress induction. However, MBSR-T did not seem to substantially impact self-reported mindfulness or resilience, nor the expression of pro-inflammatory cytokines (IL-6 and CRP) or HPA axis regulatory genes (FKBP5). Taken together, the results show some evidence for efficacy on a symptom level but not on a biological level.

A number of cognitive interventions have successfully reduced symptoms of depression, anxiety, and PTSD in youth exposed to ELS [46]. However, many of these interventions are focused on specific diagnoses and/or symptom profiles. MBSR-T offers a transdiagnostic treatment approach that can be more easily disseminated in group-based settings within schools or community programs. Meta-analyses cite mindfulness-based therapies as promising interventions for treating mood and anxiety disorders in clinical adult populations [84] and adolescents and young adults [85]. Our study extends that research by providing evidence that MBSR-T is a feasible and acceptable transdiagnostic intervention for ELS-exposed youth. The completion rate (76%) and homework compliance ratings (50%) were similar to that reported in other studies utilizing psychological intervention approaches for adolescents or young adults [86, 87] and working alliance was similar to that reported in validation literature [64].

Our findings further replicated previous research utilizing MBSR-T in other adolescent populations [88, 89], by demonstrating that MBSR-T may also be effective in reducing symptoms of depression for ELS-exposed youth. This is particularly promising given that the youth in this study were not identified based on elevated depression symptoms, suggesting that it may be beneficial even when delivered to youth without diagnosable mental health disorders. MBSR-T may be particularly helpful for reducing depression and related outcomes for adolescents with ELS, as mindfulness promotes noticing and regulating self-referential maladaptive thoughts, emotional responses, and behaviors often precipitating and perpetuating mental health conditions [90]. However, further research is needed to delineate the essential mechanisms and long-term impact.

This feasibility study with adolescents with ELS exposure supports previous research that links mindfulness and changes in responses to stress induction [55]. Indeed, participants in the MBSR-T group showed an intervention effect on both self-report and salivary cortisol responses. Relative to CTRL, MBSR-T participants evidenced lower levels of cortisol in anticipation of the TSST-C, and increased cortisol reactivity following the TSST-C at follow-up. This may indicate a more adaptive physiological stress response [91]. Further, MBSR-T participants reported less anxiety as a function of stress induction relative to CTRL, which has been demonstrated in previous research [92]. We posit that MBSR-T participants were able to recruit adaptive stress-reduction techniques discussed in the intervention in response to stress induction. Taken together, the stress reactivity mechanism may be one pathway through which mindfulness techniques exert benefits for trauma-exposed youth and sub-clinical populations.

Although there is preliminary data to suggest that mindfulness practice in adults may positively influence processes involved in regulation of stress responses, including immune and endocrine system markers [54, 55] and gene expression [52], we were unable to detect these differences in neurobiological markers of interest in our adolescent sample. Previous studies have shown these effects in community samples, expert mediators, or over longer period of time and greater number of sessions. It is possible that the length of intervention in this study was too short to exert and detect changes in endocrine and epigenetic systems, particularly in a young population with ELS exposure. Indeed, extant literature suggest that interventions for trauma-exposed individuals may need to be adapted (i.e., lengthened) to account for dysregulated systems [93]. Nevertheless, it is well known that ELS exposure during critical periods of development alters the functioning of the brain, endocrine, and immune systems involved in regulation of stress response [94–97], in turn accounting for the observed short- and long-term negative mental and physical outcomes. Therefore, interventions that can reverse or compensate for these neurobiological disruptions are needed in order to optimize functioning and outcomes in these populations. To see more of an impact on these neurobiological systems, MBSR-T may be enhanced with other psychological (e.g., cognitive restructuring), neuromodulatory (e.g., real time functional neuroimaging neurofeedback) techniques, and the use of smart-phone technology (e.g., ecological momentary assessments). These enhancements are poised to supplement the dosing of mindfulness and other cognitive techniques, more effectively engage in and self-regulate brain activity, and increase between-session compliance through self-monitoring, respectively. Future studies with larger samples and greater variability in trauma exposure and symptomatology will be needed to further establish potential changes in these systems and more definitively determine whether mindfulness interventions can exert changes at the neurobiological level for adolescents with ELS exposure.

### Limitations and future research

This feasibility study possesses many strengths, including the use of a control condition, randomization, and multi-level assessment of a mindfulness-based intervention in adolescents exposed to ELS. However, it is not without limitations. Primarily, we are limited by a small sample size. Large sample sizes are often required to detect differences in biomarkers, particularly of genes. Additionally, the lack of a standardized blood and saliva collection time introduced natural variability, and future larger studies will benefit from standardized and more comprehensive collections. We examined very few biological markers, limiting the conclusions we may draw on the potential effect mindfulness interventions may have on immune and endocrine systems. Ongoing research is needed to delineate the impact of specific types of abuse (e.g., threat, deprivation) on biological systems, as well as how might they be differentially impacted by mindfulness interventions. Next, in an effort to increase generalizability, we included adolescents impacted by ELS, regardless of the presence of mental health symptoms. Larger studies would be able to maintain this approach while allowing for sampling of participants with greater symptom severity and examine the extent MBSR-T may exert over symptom reduction in a clinical population. Further, the use of a non-treatment control group could be enhanced by the use of an active control, such as Health Enhancement Programs [98], to further delineate mindfulness as the mechanism of change observed in these data. Next, the delivery of the intervention was not assessed for fidelity by a third party. However, expert consultation was provided to prevent or correct for any potential bias introduced by investigators leading the intervention. Finally, longitudinal studies will be able to examine whether these interventions increase resilience in the long-term among youth affected by ELS exposure.

## Conclusions

The current feasibility study presented results from a randomized controlled trial of a brief mindfulness intervention program (MBSR-T) for adolescents exposed to ELS. This intervention had a positive impact on self-reported symptoms of depression as compared with adolescents in the control group. Therefore, the results demonstrate that mindfulness-based interventions for adolescents exposed to ELS are safe, feasible, and aid in reducing depressive symptoms. MBSR-T also demonstrated changes in stress perception and regulation, indicating that stress reactivity may be one key mechanism underlying the positive effects of MBSR-T. Thus, we propose group-based MBSR-T as a valid format for adolescents with exposure to early life stress. Still, future studies should leverage larger as well as more clinically diverse adolescent populations and examine the effect of mindfulness interventions on neurobiological functioning in this population. The effect on outcomes of moderating factors such as age, gender, type and severity of symptoms, previous and current use of psychotherapy and/or pharmacotherapy treatment should be examined. Finally, augmentation techniques, such as ecological momentary assessments and feedback and real-time functional magnetic resonance imaging neurofeedback (rtfMRI-nf), are poised to enhance mindfulness training and practice.

## Supplementary Information

**Additional file 1: Figure S1.** The trajectory of the total score of the Working Alliance Inventory. Therapeutic alliance evidenced minimal changes over the course of treatment among both the total scores and subscale scores. **Table S1**. Schedule of events for self-report measures. **Table S2**. Multiple regression analyses of baseline depression symptoms and biomarkers. **Table S3**. Homework rating scale within the mindfulness-based stress reduction for teens group. **Table S4**. Working Alliance Inventory within the mindfulness-based stress reduction for teens group.

## Data Availability

The datasets used and/or analyzed during the current study are available from the corresponding author on reasonable request.

## Abbreviations

ACEs: Adverse Childhood Experiences scale
AADIS: Adolescent Alcohol and Drug Involvements Scale
B: Baseline
CD-RISC 10: Connor-Davidson Resilience Scale, 10 item
CTQ: Childhood Trauma Questionnaire
CTRL: Control
F/U: Follow-Up
MAAS-A: Mindful Attention Awareness Scale for Adolescents
MFQ: Mood and Feelings Questionnaire
OA: Online Assessment
S: Session
Scrn: Screening
TSST-C: Trier Social Stress Test for Children
