# Supplementary material for: The effect of a mindfulness-based stress intervention on neurobiological and symptom measures in adolescents with early life stress: a randomized feasibility study
Source: BMC Complement Med Ther. 2021 Apr 15;21:123. doi: 10.1186/s12906-021-03295-1 (PMC8050904; doi:10.1186/s12906-021-03295-1)
Supplement: Supplementary file 1 — Additional file 1: Figure S1. The trajectory of the total score of the Working Alliance Inventory. Therapeutic alliance evidenced minimal changes over the course of treatment among both the total scores and subscale scores. Table S1. Schedule of events for self-report measures. Table S2. Multiple regression analyses of baseline depression symptoms and biomarkers. Table S3. Homework rating scale within the mindfulness-based stress reduction for teens group. Table S4. Working Alliance Inventory within the mindfulness-based stress reduction for teens group. [file 12906_2021_3295_MOESM1_ESM.pdf]

The effect of a mindfulness-based stress intervention on neurobiological and symptom measures in adolescents with early life stress: A randomized feasibility study

Zsofia P. Cohen, BS[1], Kelly T. Cosgrove, MA[1,2], Elisabeth Akeman, MS[1], Sara Coffey, DO[3], Kent Teague, PhD[3,4], Jennifer Hays-Grudo, PhD[3], Martin P. Paulus, MD[1], Robin L. Aupperle, PhD[1,5], and Namik Kirlic, PhD[1*]

[1]Laureate Institute for Brain Research, Tulsa, Oklahoma
[2]Department of Psychology, University of Tulsa, Tulsa, Oklahoma
[3]Center for Health Sciences, Oklahoma State University – Tulsa, Tulsa, Oklahoma
[4]Departments of Surgery and Psychiatry, University of Oklahoma School of Community Medicine, Tulsa, Oklahoma
[5]School of Community Medicine, University of Tulsa, Tulsa, Oklahoma

*** Correspondence:**
Namik Kirlic, PhD
Associate Investigator
Laureate Institute for Brain Research
6655 South Yale Ave
Tulsa, OK 74136
Phone: 918-502-5747
Email: nkirlic@laureateinstitute.org

Supplementary Tables

Table S1. Schedule of events for self-report measures.

Measure	SCRN	BASE	OA1	OA2	S1	S2	S3	S4	S5	S6	S7	S8	F/U
ACEs	·	·											·
AADIS		·											·
CD-RISC 10		·											·
CTQ		·											·
MAAS-A		·	·	·									·
MFQ		·	·	·	·	·	·	·	·	·	·	·	·
Homework Rating Scale						·	·	·	·	·	·	·	
Working Alliance Inventory						·	·	·	·	·	·	·	

Note. Time between pre-intervention assessments and the start of MBSR-T or CTRL took an average of 15.44 days [SD = 13.78]. MBSR-T or CTRL was delivered over an average of 27.52 days [SD = 3.31]. Time between completing intervention and post-intervention assessments took an average of 10.38 days [SD = 4.58].

Abbreviations: ACEs, Adverse Childhood Experiences scale; AADIS, Adolescent Alcohol and Drug Involvements Scale; B, Baseline; CD-RISC 10, Connor-Davidson Resilience Scale, 10 item; CTQ, Childhood Trauma Questionnaire; CTRL, Control; F/U, Follow-Up; MAAS-A, Mindful Attention Awareness Scale for Adolescents; OA, Online Assessment; S, Session; Scrn, Screening; MFQ, Mood and Feelings Questionnaire.

Table S2. Multiple regression analyses of baseline depression symptoms and biomarkers.

	R^2	B	SE B	β	p
Cortisol	0.315				0.10
CTQ		-0.15	0.20	-0.15	0.46
Gender		-0.13	0.43	-0.06	0.77
BMI		-0.26	0.21	-0.23	0.23
Wakeup Time		0.10	0.15	0.15	0.48
Time of Collection		-0.14	0.06	-0.44	<.05
CRP	0.373				<.005
CTQ		-0.23	0.24	-0.23	0.13
Gender		-0.30	0.31	-0.15	0.33
BMI		0.68	0.16	0.64	<.001
IL-6	0.355				<.005
CTQ		0.03	0.19	-0.09	0.90
Gender		-0.04	0.25	-0.02	0.93
BMI		0.55	0.13	0.65	<.001
Depression	0.02				0.25
CTQ		0.25	0.18	0.26	0.12
Gender		-0.89	1.82	-0.08	0.63
FKBP5	0.003				0.39
CTQ		0.25	0.29	0.24	0.91
Gender		-0.24	0.38	-0.12	0.51
BMI		0.18	0.19	0.18	0.29

Abbreviations: BMI, Body Mass Index; CTQ, Childhood Trauma Questionnaire; CRP, C-reactive protein; IL-6, interleukin-6.

Table S3. Homework rating scale within the mindfulness-based stress reduction for teens group.

Session	Mean	SD	Range
1	-	-	-
2	25.15	6.56	9.00 - 40.00
3	25.31	13.95	0.00 - 40.00
4	24.24	8.18	2.00 - 35.00
5	23.00	8.54	0.00 - 36.00
6	23.40	10.57	0.00 - 42.00
7	21.67	10.17	0.00 - 37.00
8	23.14	12.09	0.00 - 40.00

Note. Rated on a 5-point Likert scale (0 = not at all, 4 = completely), possible scores range from 0 to 48.

Table S4. Working Alliance Inventory within the mindfulness-based stress reduction for teens group.

	Goals			Tasks			Bond			Total		
Session	Mean	SD	Range	Mean	SD	Range	Mean	SD	Range	Mean	SD	Range
1	-	-	-	-	-	-	-	-	-	-	-	-
2	14.41	2.94	10.00 - 20.00	14.24	2.61	9.00 - 20.00	14.71	3.14	10.00 - 20.00	43.35	7.42	32.00 - 60.00
3	15.00	4.10	4.00 - 20.00	14.40	4.02	6.00 - 20.00	15.50	4.59	4.00 - 20.00	44.90	12.01	14.00 - 60.00
4	14.41	4.51	4.00 - 20.00	14.35	3.37	8.00 - 20.00	15.53	3.52	11.00 - 20.00	44.29	10.15	28.00 - 60.00
5	14.67	3.79	7.00 - 20.00	13.67	2.66	10.00 - 20.00	15.00	3.27	12.00 - 20.00	43.33	8.84	31.00 - 60.00
6	15.36	4.78	4.00 - 20.00	15.36	3.61	9.00 - 20.00	16.21	3.83	10.00 - 20.00	46.93	10.95	31.00 - 60.00
7	15.08	3.96	8.00 - 20.00	15.75	3.47	9.00 - 20.00	16.25	3.93	8.00 - 20.00	47.08	9.85	27.00 - 60.00
8	14.79	4.79	8.00 - 20.00	15.79	3.83	8.00 - 20.00	15.14	4.64	8.00 - 20.00	45.71	12.42	24.00 - 60.00

Note. Rated on a 5-point Likert scale (1 = seldom, 5 = always), possible scores range from 4 to 20 for each subscale score and 12 to 60 for the total scale score.

Supplementary Figure

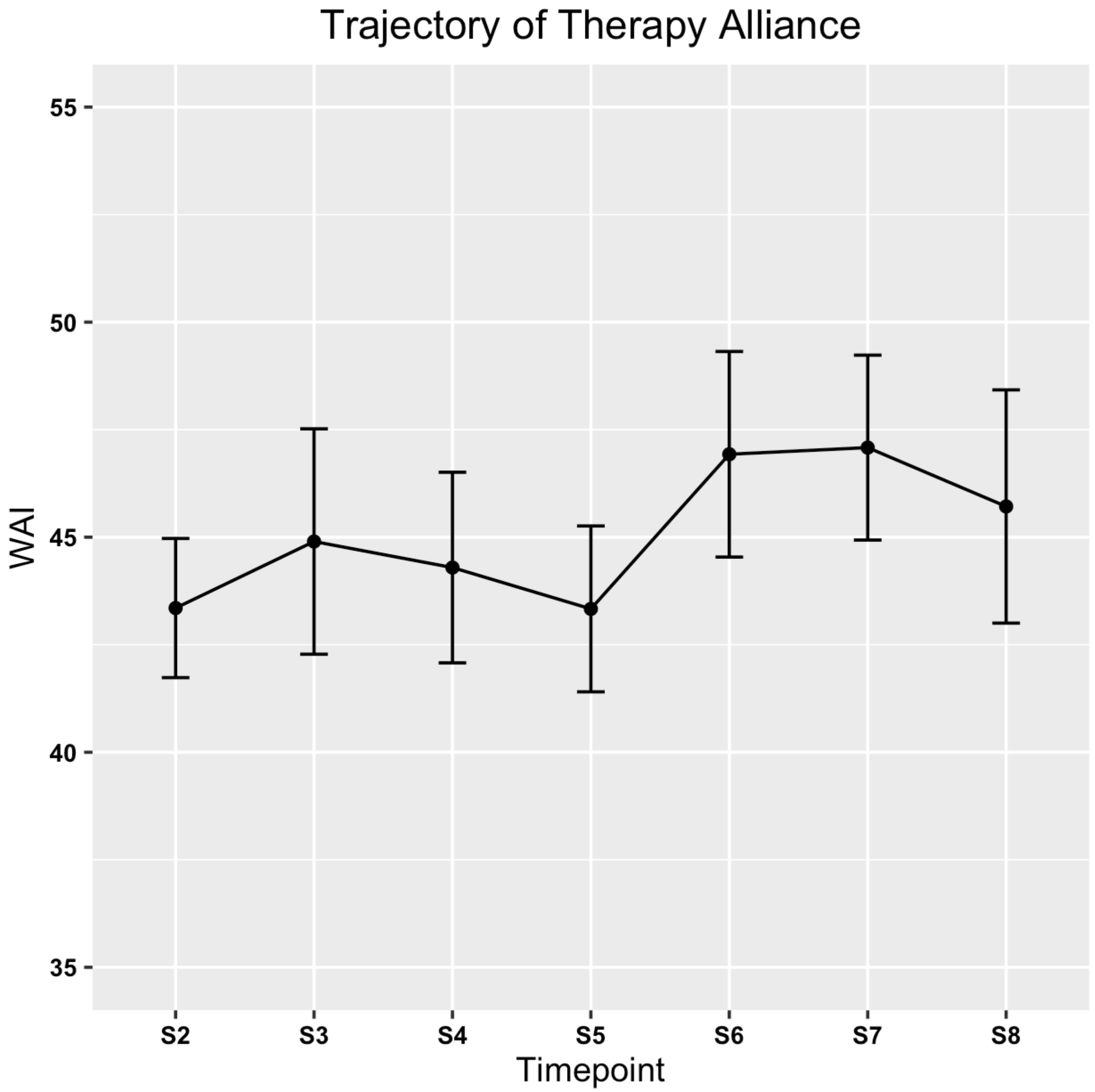

Figure S1. The trajectory of the total score of the Working Alliance Inventory. Therapeutic alliance evidenced minimal changes over the course of treatment among both the total scores and subscale scores.
